# Pellagra

**Published:** 1910-10

**Authors:** Thos. F. Taylor

**Affiliations:** Tuskegee, Ala.


### PELLAGRA.

BY THOS. F. TAYLOR, TUSKEGEE, ALA.

I shall not attempt to enter into a labyrinth of detail as to the history of pellagra, for all of you are more or less familiar with that part of it, and we are, or should be, more interested in the other phases of the subject. Suffice it to say, that it was until recently almost entirely of European habitat, and was confined in the main to Italy's borders, slaying her population with a ruthless hand.

The paper which I shall present is partially from my own observation and experience, and partially what I have culled from leading authorities on this important disease. I shall not attempt to give you anything alarmingly new, but merely to give expressions of my own opinions and the concensus of opinion of some of the best authors, hoping that I will be able to elicit a free discussion pro and con in order that we may arrive at the best possible result.

I shall define pellagra as a non-contagious, systemic toxicosis, occurring for the most part during the spring and summer months, characterized by a triad of symptoms, viz: stomato-gastro-intestinal, nervous and cutaneous, whose specific causative factor is not yet definitely determined.

### Etiology.

As to the etiology of this disease, the research men are divided between the maize theory and the protozoan theory, with by far the greater number in favor of the former,—and in the light of our present knowledge, I am persuaded that the corn or maize theory of causation is by far the strongest one to hold to, for the reason that it has its greatest prevalence in corn consuming if not corn producing districts, and for the most part among classes who employ corn-meal as the chief article of diet on account of financial reasons.

It is my opinion, too, that this toxin from the immature or ill-cured corn may be taken into the body and there lie dormant for months or even years, so long as the patient has sufficient vitality to overcome it, and that just as soon as there is a sufficiently diminished physiological resistance, this toxin asserts itself, as do other diseases, with a severity proportional to the virilence of the poison and the resistance of the tissues.

The adherents of the protozoan theory have, however, given some able argument, for instance, the occurrence of the disease in infants who are nourished entirely from the breast. But may we not assume that the poison is transmitted from the mother, even though she has not developed the disease? Then, too, sporadic cases of many diseases occur here and there without determined traceable causes. The adherents of this theory have spent more time trying to disprove the diseased corn theory than towards establishing their own theory.

The majority of cases in this country have been reported from insane hospitals, and we are told by the authorities there that when the inmates are taken off corn or meal diet the number of cases diminish, which, to my mind, is rather a strong argument in favor of the maize theory.

### Symptomology.

While most of us are more or less familiar with the symptoms of the disease, I think they cannot be rehearsed too often so that we may be thoroughly familiar with them in order that we may be able to recognize the early cases.

Like nephritis, and some other diseases, pellagra has gotten in its mischief before the cases come under the observation of the physician, another instance where it is "too late for the balm when the heart is broken."

It has not been my good fortune to get hold of many incipient cases, consequently I cannot speak personally on the symptomatology of such cases, but most authors describe them about as follows: Lassitude, general disinclination to work, indigestion, tired feeling, headaches, and occasional diarrhoea.

Typical cases present the following symptoms: As a rule in the cases I have seen, the gastro-intestinal trouble makes its appearance first, though one case I recall had well developed mania several months previous to a pellagra diagnosis. Temperature usually around 99 to 100 degrees F., pulse slightly accelerated. The tongue becomes very red, almost scarlet in some, the gums swollen, red and spongy. Salivation is profuse, the patient constantly expectorating large quantites of more or less viscid saliva having a peculiar offensive odor. Later the tongue is denuded of epithelium, and presents a glazed appearance. Nausea and anorexia are common. Thirst is marked, but very probaably nature's method of supplying fluid lost by excessive expectoration and diarrhoea.

Diarrhoea ofttimes is extreme, the patient passing ten to thirty watery stools in 24 hours. In some cases constipation is the rule, but such cases are rare. Others alternate with diarrhoea and constipation. The diarrhoea, in my opinion, is more or less compensatory, being nature's method of getting rid of the poison. For this reason it should not be checked entirely, as by giving strong astringents. Five to eight stools in 24 hours are not too many, and should not be reduced below this number. Usually a non-compensatory diarrhoea can be checked by free saline purges and high boric acid enemas, but not so with pellagrous diarrhoea, colon flushings and intestinal sweepings are of no avail.

The whole gastro-intestinal tract from mouth to anus is inflamed, and it would probably be fair to state that there is a denudation of the endothelial lining of the entire tract. For this reason the patient is unable to absorb sufficient nourishment to keep up metabolism aside from the debilitating toxaemic condition. Exhaution being due ofttimes as much to the lack of absorbing and assimilating sufficient food, as to the direct inroads of the disease per se. The patients rarely complain of much pain, this being in a measure due to the lowered mental state.

In from two to six weeks the cutaneous changes occur. They have been very aptly described as having the appearance of sunburn. There is an eruption occurring on the dorsal surfaces of the hands and wrists extending to within two or three inches of the elbow. Likewise the feet and legs. I have seen several cases where the eruption was present on the palma and plantor surfaces as well. The back of the neck, forehead, maler prominences, vaginal and scrotal regions often being involved. Indeed, I have examined cases in which the vaginal lining was as red as the tongue, and excreted a similar offensive discharge to the saliva. The exposed surfaces suffer most, being aggravated by the actinic solar rays. The sun's rays aggravate, but do not seem to be necessary to the cutaneous lesions. The lesions first appear as an erythematous rash, invading the epidermis. This gradually increases in redness till it begins to desquamate, sometimes forming blebs which break and peel off, leaving, in mild cases, almost normal skin. The severer cases, which are in the majority, involve the derma, the epidermis turns dark and has a charred appearance, afterwards scaling off, leaving a raw, weeping, red surface, which is sometimes long in healing, and leaves the skin dark and shiny.

The nervous and mental symptoms are very important, for we have to look forward to sending our patients to insane hospitals in spite of all we can do to the contrary. I have never seen a case of any standing in which the cerebro-spinal system was not affected. This usually comes on after the disease is well established, but may occur even before any pellagra symptoms are noted. I do not agree, however, with those who have suggested that insanity predisposes to the disease, but am rather of the opinion that insanity is an accompanying symptom, or rather an end-product of the disease. The mental aspects are, as a rule, of a melancholia type, gradually relapsing into secondary or terminal dementia, some have maniacal outbreaks and others take on a form of circular insanity, if indeed, they last long enough to "go the rounds."

Delusions of persecution are much in evidence, the patient often thinking he has been poisoned, tricked, "hoodooed," and the like. Those few cases of the maniacal type die earlier, being assisted by the exhaustion. The tendon reflexes towards the beginning are exaggerated, but after the disease gets well under way, they gradually become diminished and are eventually abolished. I have not noticed much pupillary change.

### Diagnosis.

The diagnosis in typical cases can hardly be mistaken after having seen a well developed case, the gastro-intestinal, nervous and cutaneous changes being pathognomonic. Atypical cases, without the eruption, are apt to be confounded with idiopathic melancholia or dementia, with accompanying auto-toxicosis.

Scurvy may be eliminated after the cutaneous lesions appear. The erythemas usually can be excluded, as they are wanting in the mental and characteristic gastro-intestinal symptoms.

### PATHOLOGY.

In looking over the pathology, as given by various authors, I observe the findings to be quite diversified, showing that no fixed rule can be laid down in regard to the organic changes. All, however, are agreed on an enlarged nutmeg liver, atrophy of viscera supplied by the vagus. Hyperaemia or intestines leading to denudation of epithelium. Pachymenengitis, slight degenerative changes in brain and cord,—other than this there seems to be no definite pathology.

### PROGNOSIS.

The prognosis in acute cases is death from three weeks to two months under most any kind of treatment. Mild cases recover temporarily with the approach of cold weather only to recur the following spring. This may go on for a number of years, each attack making the patient more demented and debilitated, finally terminating in death with pellagra, either directly or indirectly as the cause.

### TREATMENT.

The treatment resolves itself into prophylactic and symptomatical. The prophylactic is far more important, and if persisted in, will reap its reward. It consists in, in recurrent cases, the building up of the general physical condition early every spring, by means of iron, arsenic and nourishing foods other than of corn origin. This, of course, is to build up the physiological resistance in order to ward off the coming danger. For the primary cases, we know of nothing better just now than to advise against the use of corn bread until we inaugurate better methods of curing corn and corn products for food consumption.

The drug treatment consists in the main of arsenic, preferably in form of Fowler's Solution, 5 to 15 drops three times during the 24 hours. Salol grs. 5, tid makes a good intestinal antiseptic. The mouth should be kept clean by constant use of euthymol or listerine, or some such wash.

When the diarrhoea is too profuse, it should be partially checked, bismuth, T. R. opium and zinc salts preferred. The erythematous surfaces should be frequently cleaned, dusted with boric acid and kept bandaged.

Transfusion has been done in a few cases with apparent success. It is only recommended, however, in extreme cases and as a last resort. Of the reports noted, many of the benefitted cases received blood from donors who had not previously had the disease, suggesting that the benefit is due to a supplanting of the anaemia rather than supplying an anti-body.

